# Development and Application of the Placebo Response Model in Clinical Trials for Primary Sjögren’s Syndrome

**DOI:** 10.3389/fimmu.2021.783246

**Published:** 2021-11-16

**Authors:** ZHI-Zhou Wang, Qing-Shan Zheng, Hong-Xia Liu, Lu-Jin Li

**Affiliations:** Center for Drug Clinical Research, Shanghai University of Traditional Chinese Medicine, Shanghai, China

**Keywords:** Sjögren’s syndrome, placebo response, EULAR Sjögren’s syndrome disease activity index (ESSDAI), Model-based meta-analysis, simulation

## Abstract

This study aimed to develop a placebo response model for pharmaceutical clinical trials of primary Sjogren’s syndrome,and to quantitatively analyze the distribution and related factors influencing the placebo response to further optimize the design of clinical trials and evaluate the results of single-arm clinical trials. Public databases, including PubMed, Embase, and Cochrane Library were searched for reports on randomized placebo-controlled trials for Sjögren’s syndrome which used the change from baseline in ESSDAI score as the primary outcome. The model-based meta-analysis method was used to evaluate the time course and the related influencing factors of the placebo response for ESSDAI in such clinical trials. A virtual placebo control group was constructed based on the final placebo response model to determine the treatment efficacy of belimumab and cyclosporine A for primary Sjögren’s syndrome in a single-arm study. A total of 12 studies involving 450 subjects were included in the analysis. The established model described the time-course characteristics of the changes in ESSDAI score from the baseline in the 48 weeks placebo group. We found that the onset time of placebo response was approximately 12 weeks, and its efficacy plateaued at 48 weeks. The baseline ESSDAI score had a significant effect on the maximum value of the placebo response; the maximum value of the placebo response decreased by 0.552 for every 1 score rise in the baseline ESSDAI score. The efficacy of belimumab and cyclosporine A in the single-arm trial was comparable to that of the placebo response at the same baseline; no significant therapeutic advantage was observed. The placebo response model established in this study could provide a basis for designing clinical trials for primary Sjogren’s syndrome in the future. It may also provide a reliable external efficacy control standard for single-arm clinical trials.

## Introduction

Sjögren’s syndrome (SS) is a chronic inflammatory autoimmune disease that primarily affects exocrine glands. With a prevalence of 0.1% to 4.8% (1, 2), it mainly affects adult female patients (3). The common clinical manifestations of Sjögren’s syndrome include ocular and oral dryness, fatigue, and joint pain (4). In addition, 5%–50% of patients have systemic symptoms in the lungs, kidneys, blood vessels, skin, and nervous system (5, 6), which can seriously impair patients’ quality of life (7).

Simple Sjogren’s syndrome without other associated connective tissue diseases is known as primary Sjogren’s syndrome (PSS) (8). The therapeutic management of primary Sjögren’s syndrome has not changed substantially in the last few decades (9). Therapeutic goals focus on relieving the symptoms, preventing systemic damage, and improving the patient’s quality of life. Treatment options can either be local or systemic therapies. The drugs currently used for systemic therapy include glucocorticoids, immunosuppressive agents, and biological agents (10, 11). Previous studies show that the therapeutic effects of these drugs are not prominent (12, 13); therefore, it is necessary to develop safer and more effective drugs for the treatment of PSS.

Previous meta-analyses show that the efficacy of most drugs in randomized controlled trials (RCTs) for PSS are not significantly different as compared to the placebo administration (14, 15). Due to the high placebo response and extensive inter-trial variations in such clinical trials (12), it is difficult to evaluate the effectiveness of new drugs. Therefore, understanding the distributional characteristics and factors influencing placebo response in clinical trials of PSS are critical for designing the clinical trial and judging the abnormal placebo response. However, only a few studies have systematically evaluated the effect distributional profile of placebo in pharmacotherapy for PSS.

In recent years, the EULAR Sjögren’s Syndrome Disease Activity Index (ESSDAI) has been widely used in clinical studies as inclusion criteria and to define endpoints (16). Within ESSDAI, the clinical or biological manifestations are divided into 12 domains and scored separately; each domain’s score is also stratified according to the severity of the manifestations. The maximum theoretical ESSDAI score is 123 points, and minimal clinical improvement is defined as an improvement in at least three points (17). In this study, a pharmacodynamic model was established using model-based meta-analysis (MBMA) (18, 19) to quantitatively analyze the placebo response distributional characteristics and factors influencing the ESSDAI parameters in PSS trials. The findings may provide an effective reference for drug development and clinical trial design for PSS. The study may also provide reliable external control for single-arm clinical trials for pharmacotherapy in PSS.

## Methods

### Search Strategy

The literature search was performed using three public databases, PubMed, Embase, and the Cochrane library; relevant publications till May 28, 2021, were included. The search keywords were “Sjögren’s Syndrome” and “placebo.” Entries of the same category were connected with the logical word “or”; entries of different categories were connected with the logical word “and”. Clinical trials were searched, and the language of publication was limited to English. Detailed search strategies are provided in the **Supplementary Material**. In addition, manual searches were performed from the references of the relevant review articles to include all trials which were potentially missed during the database search.

### Inclusion and Exclusion Criteria

The inclusion criteria were as follows: (1) randomized placebo-controlled trials, (2) adult patients diagnosed with PSS, (3) pharmacotherapeutic intervention, and (4) the baseline ESSDAI score and the ESSDAI score at different time points were provided.

To avoid the legacy effect, only data from the first treatment period were included for the present analysis and the crossover trial design. To reduce heterogeneity across trials, studies on subjects with secondary Sjögren’s syndrome were excluded.

### Data Extraction

The WPS Excel software (11.1.0.9914) was used as a template for database entry. The following information from the literature that met the inclusion criteria was extracted: literature characteristics (literature ID, authors, year of publication, and country), trial design (trial type, group, treatment duration, test drug, and sample size), characteristics of subjects (age, proportion of male subjects, time since diagnosis, baseline ESSDAI score, and treatment history), and trial outcomes (change from the baseline ESSDAI score at each follow-up time point). When multiple analysis set results were reported for efficacy, the data of the intention-to-treat (ITT) set were preferred. All of the above information was extracted independently by two investigators, and inconsistencies were resolved jointly by a third investigator. If the efficacy data in the literature were presented as a graph, a digitizing software, GetData Graph Digitizer (2.25.0.32) was used for data extraction. If the data extraction error between the two investigators exceeded 2%, the data extraction was repeated.

### Risk of Bias Assessment

The Cochrane risk-of-bias table was used to evaluate the quality of the literature (20). The evaluation items included random sequence generation, allocation concealment, blinding of participants and personnel, blinding of outcome assessment, incomplete outcome data, selective reporting, and other biases. The term”other bias”was defined as the sponsorship of the trial by drug companies, early termination of the trial, and incomparable baseline for subjects in each trial group. The quality of each item was graded as low, high, or unclear. Two investigators independently scored the quality of the literature, and a third investigator adjudicated any discrepancies.

### Model Development

Exploratory analysis of the data showed that the placebo responses in the PSS clinical trials gradually increased over time and eventually plateaued. The distributional characteristics of these data could be described by the Emax model [see Eq. (1)]. This model had two important parameters: Emax and ET50. Emax represented the maximum effect, and ET50 was the onset time. The base model was described as follows:


(1) $$E_{\text{ij}}=-\frac{E_{\max,i}\times Time_{j}}{ET_{50,\text{i}}+Time_{j}}+\frac{\epsilon_{i,j}}{\sqrt{N_{i,j}}}$$

(2) $$P_{i}=P_{typical}+\eta_{i}$$

(3) $$P_{i}=P_{typical}\times e^{\eta_{i}}$$

In Eq. (1), Ei, j is the effect at the observation time point j of the trial i placebo group; Emax, i is the maximum placebo response of trial i; ET50, i is the time taken for the trial i placebo group to achieve half the Emax; Time represents the observation time (week); is the residual error at the observation time point j of the trial i placebo group; Ni, j is the sample size at the observation time point j of the trial i placebo group, needs to be corrected by the inverse of the square root of the sample size; that is, the larger the sample size, the smaller the residual value. was assumed to be normally distributed with a mean of zero and a variance of. is the inter-trial variability for pharmacodynamic parameters and was added to the parameters Emax and ET50 in the additive model [Eq. (2)] or exponential model [Eq. (3)], if available. was assumed to be normally distributed, with a mean of zero and a variance of.

After the base model was established, the potential factors influencing the model parameters were investigated, including age, the proportion of males, treatment duration, time since diagnosis, baseline ESSDAI score, and presence of base therapy. Covariates with ≥40% missing data were not considered, and missing values for covariates with less than 40% missing data were imputed with the median. First, the effect of covariates on the model parameters was individually investigated. If the objective function value (OFV) of the model decreased by >3.84 (df = 1, P < 0.05), the covariates were considered to have a significant effect on the parameters. All covariates with a significant influence by sieving in succession and covariates were re-screened by forward inclusion and backward elimination methods to validate the covariates that would eventually be included in the model.The cut-off value of OFV was set at 3.84 (P < 0.05) for the forward inclusion method and 6.63 (P < 0.01) for the backward elimination method (21). Detailed covariate screening process are provided in the **Supplementary Material**.

### Model Evaluation

After the final model was established, its goodness-of-fit was first evaluated using model diagnostic plots (22). The goodness-of-fit plots included the following scatter plots: observation (OBS) *vs.* individual prediction (IPRED), observation (OBS) *vs.* population prediction (PRED), conditional weighted residual errors (CWRES) *vs.* population prediction (PRED), and conditional weighted residual errors (CWRES) *vs.* time. Sensitivity analysis was performed using leave-one-out cross-validation, that is, data from one trial were removed from the original dataset each time, and the model parameters of the remaining data were estimated to investigate the effect of each trial on the model parameters (23). A visual predictive check (VPC) was used to further validate the established model, and was simulated 1000 times using the Monte Carlo method to obtain the 95% confidence interval (CI) of the model parameters; these parameters were compared with the actual observed values to assess the predictive performance of the model (24). Finally, the sampling importance resampling (SIR) method was used for repeated sampling (1000 times) to obtain the median value of the distribution of the model parameters and their 95% confidence interval, which were further compared with the respective values estimated from the final model. Thus, the robustness of the model parameters was assessed (25).

### Model Simulation

Monte Carlo simulation was used to calculate the typical value of placebo response for various influencing factors by randomly drawing values from the distribution of typical values of placebo response model parameters. This process was repeated 10,000 times to obtain the 95% confidence interval for the typical value of the placebo response under different influencing factors.

### Model Application

The efficacy of drugs for Sjögren’s syndrome in single-arm trials was evaluated based on the final placebo response model. In two single-arm trials with belimumab and cyclosporine A for the treatment of Sjögren’s syndrome (26, 27), the efficacy of 30 subjects at weeks 12, 28, and 16 were reported in each trial. The respective baseline values of ESSDAI for the two trials were 8.8 and 5.5%. Based on the placebo response model, 95% CIs were simulated for the placebo response at the same baseline as in the trial. If the observed drug efficacy data in the trial fell outside the 95% CI of the placebo response, a significant difference between the investigated drug and placebo groups was considered; otherwise, it was comparable to the placebo response.

### Software

The model was developed using NONMEM7.4 (ICON Development Solutions, USA), and the first-order conditional estimation method with interaction (FOCE-I) was selected for model parameter estimation. Model simulation and plotting were performed using the R software (version 4.0.3, The R Foundation of Statistical Computing, Vienna, Austria). Literature quality assessment was performed using the Review Manager software (version 5.4, Nordic Cochrane Center, Copenhagen, Denmark).

## Results

### Characteristics of Included Studies

**Figure 1:**
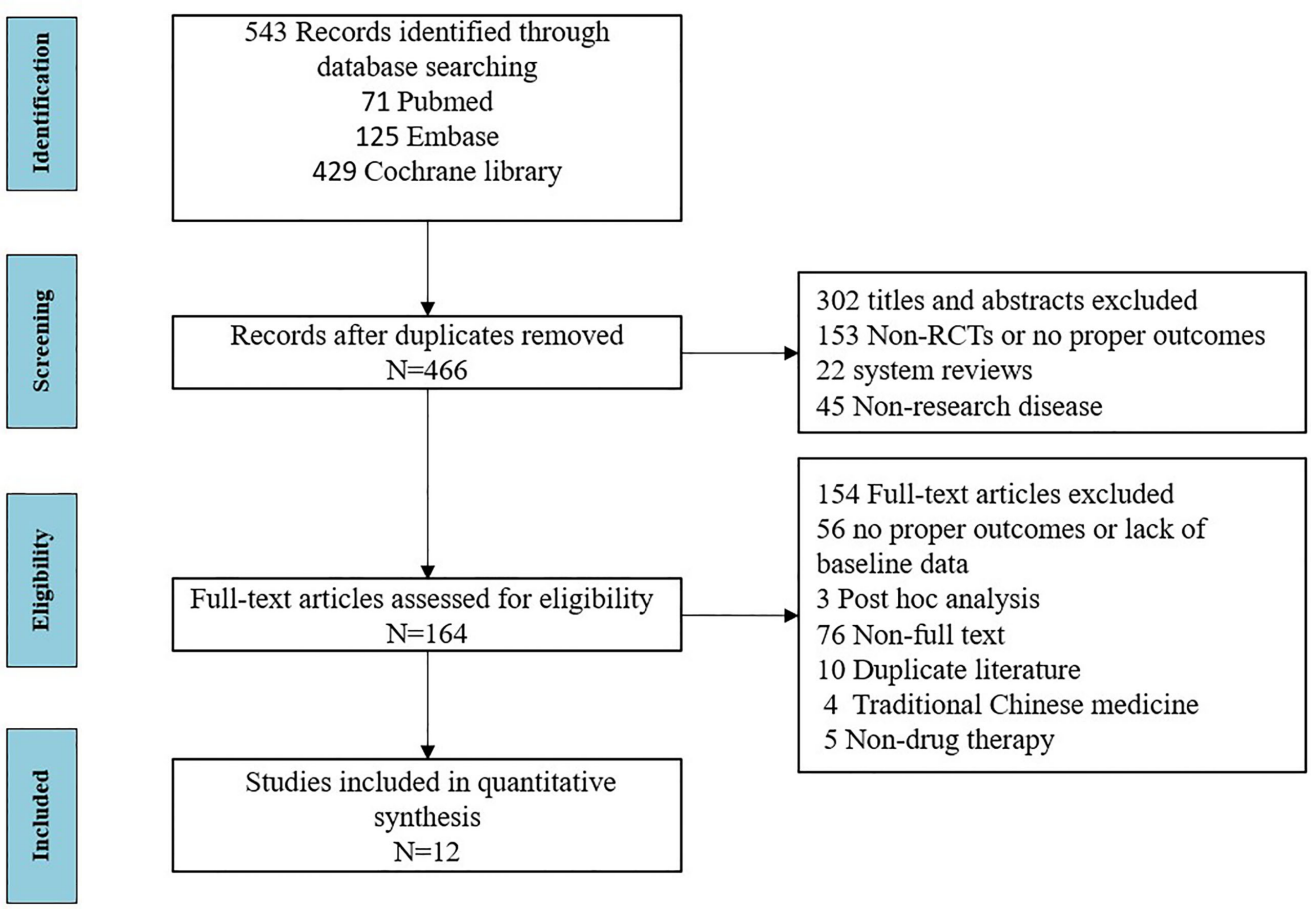
Flow chart for study identification and selection.

A total of 12 articles (28–39) published between 2014 and 2020 that met the inclusion criteria were included in the analysis, containing a total of 450 subjects from 13 trials. The detailed literature screening process is shown in **Figure 1**. The sample size of the placebo groups ranged from 4 to 95 participants (median, 19); the mean age of the subjects ranged from 48.8 to 60.2 years (median, 54.4 years); the treatment duration ranged from 12 to 48 weeks (median, 24 weeks); the percentage of male participants was between 0% and 22.2% (median, 5%); the diagnosis time ranged from 1 to 8.9 years (median, 5.3 years), and the mean baseline ESSDAI scores ranged from 2.5–13.1 (median, 10.1); the demographic characteristics of the included literature are summarized in **Table 1**. Details of the included studies are presented in **Table S1** in the **Supplementary Material**. Among the 12 included articles, the overall quality was assessed as high with a low risk of bias; detailed information on the assessment of literature quality is shown in **Figure S1**.

**Table 1:** Demographic characteristics of included studies.

	Value
Number of trials (placebo sample size)	13 (450)
Placebo sample size per arm, median (min-max)	19 (4-95)
Age,y,median (min-max)	54.4 (48.8-60.2)
Gender, male%,median (min–max)	5.0 (0-22.2)
Treatment duration,wks,median (min-max)	24 (12-48)
Time to diagnosis,y,median (min-max)	5.3 (1-8.9)
ESSDAI score at baseline,median (min-max)	10.1 (2.5-13.1)
Anti-SSA antibodies%,median (min–max)	88 (54-100)
Basic treatment%, median (min–max)	78.5 (0-100)
Abnormal SchIrmer test result,%	51.3

### Model Establishment

For the establishment of the base model, inter-study variability was added to Emax in the additive form and to ET50 in the exponential form. In the covariate analysis, only the baseline ESSDAI score had a significant effect on the Emax value. The detailed covariate screening process is presented in **Figure S2**. The final covariate model was expressed as follows:


(4) $$E_{\max}=4.44+(Ba\text{seline}-10.1)*0.552$$

According to Eq.(4), when the baseline of ESSDAI was 10.1, the Emax of the placebo response was 4.44 points, and for every 1-point increase in the baseline of ESSDAI, the Emax value increased by 0.552 points. The final estimates for the model parameters are presented in **Table 2**.

**Table 2:** Parameter estimation of the final model.

Parameters	Value	RSE (%)	SIR* median (95% CI)
E_max_(score)	4.44	14.3	4.44 (3.51,5.26)
ET_50_(week)	12.2	35.4	12.71 (7.37, 19.36)
θ(Baseline)on E_max_	0.552	18.8	0.51(0.37,0.65)
ηE_max_	0.563	24.0	0.57 (0.33, 0.74)
ηET_50_	0.794	29.4	0.80 (0.44, 1.08)
ϵ	1.661	17.3	1.69 (1.34, 2.08)

E_max_, theoretical maximum effect value; ET_50_, time to reach half of the maximum effect value; θ, covariate correction factor; η, inter-trail variability; ϵ, residual; RSE, relative standard error; CI, confidence interval; SIR* is Sampling importance resampling.

### Model Evaluation

**Figure 2:**
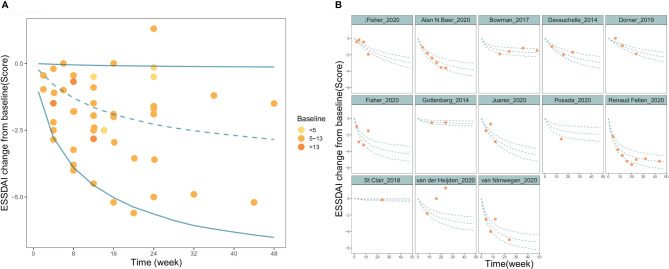
Visual predictive check of the final model. **(A)** Time course of PSS change from baseline for overall study. **(B)** Time course of PSS change from baseline for each individual study. The blue lines in the figure represent the 2.5%, 50%, and 97.5% percentiles of the placebo response predicted by the model, respectively, and the points represent the observed value.

The goodness-of-fit plots for the final model showed that the OBS, PRED, OBS, and IPRED were evenly distributed on both sides of the diagonal, and the fitted line coincided with the diagonal. For CWRES, the majority of the points were evenly distributed around the zero line within 6, and the fitted lines for CWRES *vs.* time and PRED coincided with the zero line. The above results indicated a great goodness-of-fit of the model for the actual observed values without obvious bias (**Figure S3**). Sensitivity analysis by the leave-one-out cross-validation method showed that the model parameters were not significantly affected by any individual study (**Figure S4**). The distribution of model parameters obtained by 1000 iterations in SIR was close to those obtained from the original dataset, which indicated that the estimation of the model parameters was relatively robust (**Table 2**). The visual predictive check (VPC) showed that the majority of observed data were distributed within the 95% CI of model prediction, which reflected a good prediction capacity of the model (**Figure 2**). Overall, the model evaluation results suggested that the model described the observed data reasonably well.

### Typical Placebo Response

**Figure 3:**
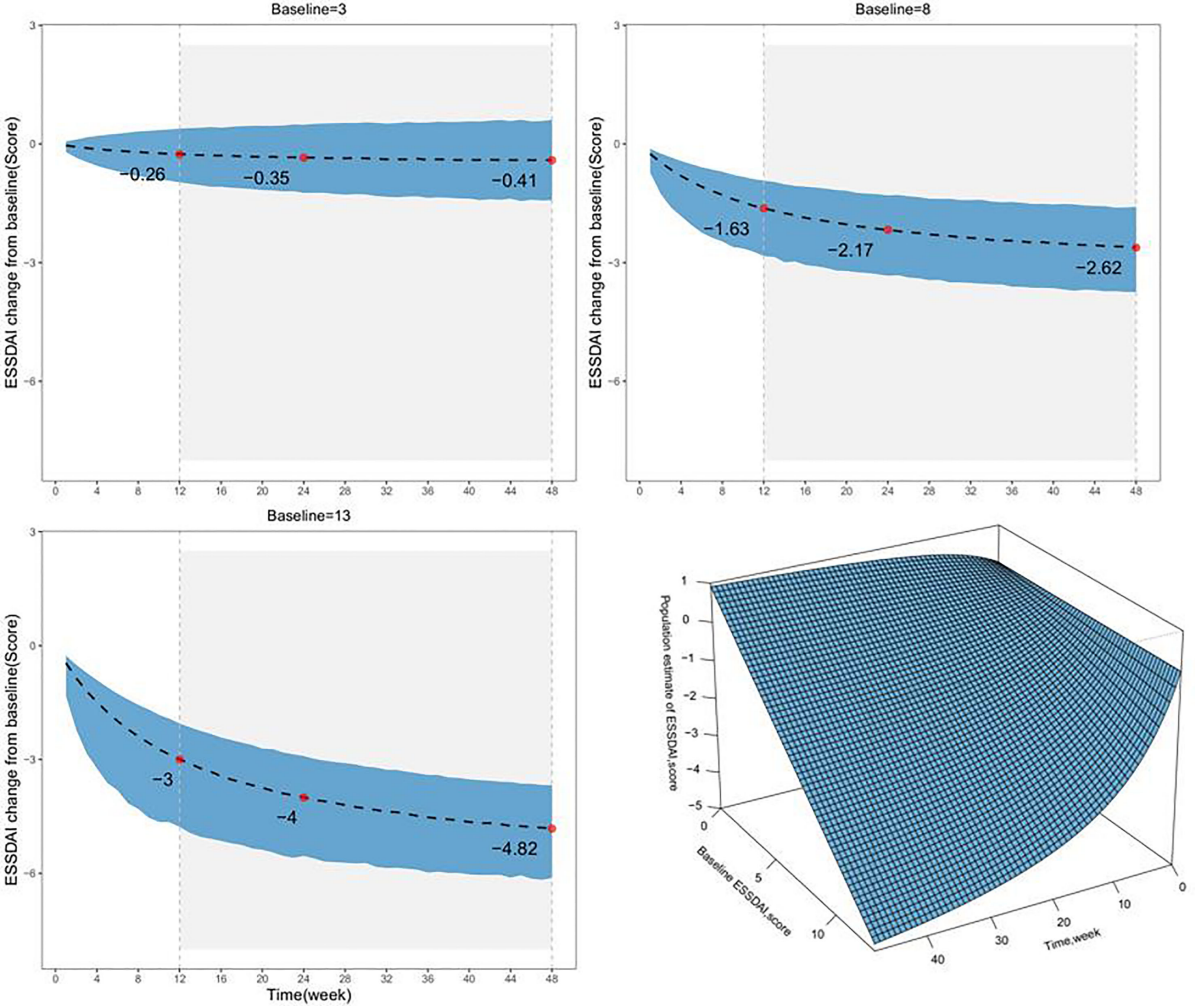
The model-estimated typical placebo time-response under different baseline levels. The dotted lines represent the typical efficacy, and the shaded areas are their 95% CIs. The points represent the estimated values of the placebo response. The lower right, a surface plot to describe the relationship among time, Baseline ESSDAI score, and the ESSDAI score. Patients with longer treatment duration and higher baseline ESSDAI score were predicted to manifest more ESSDAI score decline and better symptom relief.

The typical value of the placebo response reflects the population level estimate. Based on the final model, we simulated the distribution of typical placebo responses for different baseline ESSDAI values (**Figure 3** and **Table 3**). The results at 24 weeks showed that the median typical values of placebo response were 0.35, 2.17, and 4.00 points for subjects with baseline ESSDAI scores of 3, 8, and 13, respectively.

**Table 3:** Model-estimated typical placebo response at different time points (median, 95%CI).

Baseline (ESSDAI)	12 weeks, score	24 weeks, score	48 weeks, score
3 score	-0.26 (-0.97,0.39)	-0.35 (-1.23,0.49)	-0.41 (-1.42,0.61)
8 score	-1.63 (-2.83,-0.93)	-2.17 (-3.33,-1.30)	-2.62 (-3.74,-1.59)
13 score	-3.00 (-4.77,-2.05)	-4.00 (-5.54,-2.91)	-4.82 (-6.12,-3.67)

### Model Application

**Figure 4:**
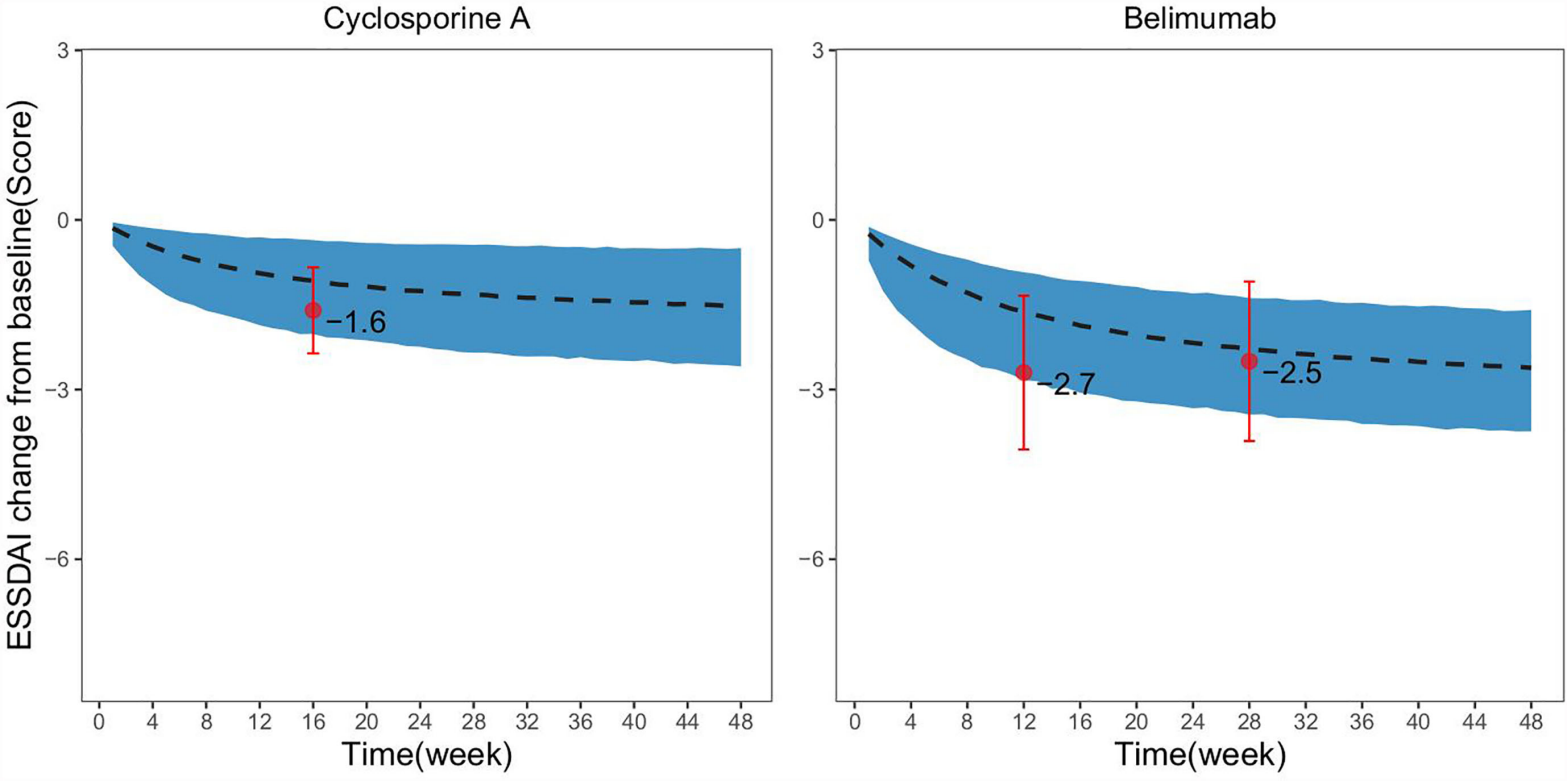
Comparison of drug efficacy and virtual placebo response distribution in single-arm trials. The dashed black line in the figure represents the typical value of the placebo response at the same baseline as predicted by the model, the shadow areas represent its 95% CI, and the points represent the actual observed values of the drug effect in the single-arm trial, the error bars represent SE for the actual observed values.

A matching placebo response distribution was simulated based on the baseline ESSDAI score for the drug group in the single-arm trial. The results showed that the observed ESSDAI values at weeks 12, 28, and 16 after administration of belimumab and cyclosporine A fell entirely within the 95% CI of the placebo response predicted by the model, which suggested that the efficacy of these two drugs were comparable to that of placebo (**Figure 4**).

## Discussion

To date, no therapy has been effective in improving the course of PSS (12). Although a few drugs used in the treatment of PSS have shown some efficacy in open-label trials, the conclusion has not been supported by high-level evidence-based medicine (40). Randomized, placebo-controlled trials are the gold standard confirmation for the effectiveness of drugs. Efficient utilization and interpretation of the placebo response from the published clinical trials of PSS are conducive for assisting the subsequent designing of clinical trials by providing a basis for the estimation of sample size, selection of treatment duration, and the establishment of inclusion and exclusion criteria for subjects. Effectively, this may reduce the sample size and improve the success rate of trials. In addition, obtaining the placebo response distribution based on the extensive literature also provides a reliable external reference for the interpretation of the results of the clinical trials and efficacy determination for single-arm trials.

In this study, based on extensive literature search and modeling, the time course and factors influencing placebo in clinical trials of PSS were quantitatively analyzed, and an accurate scale model of placebo response in PSS was constructed. The results showed that the placebo response of PSS had a significant time-course profile; the longer the duration of treatment, the more significant was the placebo response. The onset time of placebo response was approximately 12 weeks (the time to reach 50% of the maximum effect). At present, the duration of treatment in PSS clinical trials ranges from 12 to 48 weeks. In this duration of treatment, the maximum effect of placebo response was significantly different and ranged from 49.5% to 80%.It has been suggested that the heterogeneity of treatment duration between trials should be given priority when estimating the sample size of clinical trials and comparing across studies in the future.

In addition, it was found that baseline ESSDAI score had a significant effect on the placebo response; the maximum placebo response decreased by 0.552 points for every 1-point increase in baseline ESSDAI score. Considering the ethical issues, the baseline ESSDAI score in the placebo group in the included PSS clinical trials was not high and ranged between 2.5 and 13.1. In this study, the placebo response distribution of subjects with baseline ESSDAI scores of 3, 8, and 13 were simulated at different treatment durations. The placebo response of these subjects at 24 weeks was −0.35, −2.17, and −4.00 points, respectively, that is, the ESSDAI score of subjects at 24 weeks decreased to 2.65, 5.83. and 9.00 points, respectively. Thus, although the placebo response increased with the higher baseline ESSDAI score, the space left for the drug to exert its effect increased correspondingly. According to the guidelines, the minimum clinical improvement in ESSDAI score is defined as an improvement of at least 3 points (12), and the inclusion of patients with higher baseline ESSDAI levels is a prerequisite to ensure that differences between drug and placebo efficacy are observed.

The placebo response model established in this study could accurately predict the placebo response distribution of PSS clinical trials at different durations of treatment and ESSDAI baseline levels. Thus, it may provide reliable external control for other single-arm clinical trials of PSS and a basis for evaluating the efficacy of drugs. In this study, the efficacy of belimumab and cyclosporine A in PSS treatment was determined for two single-arm studies based on the established placebo response model. Belimumab, the first biological agent approved by the FDA (2011) for the treatment of systemic lupus erythematosus (SLE), is a humanized IgG1 monoclonal antibody that specifically inhibits B lymphocyte stimulating factor, thereby suppressing B cell development and maturation and inducing B cell apoptosis. Cyclosporine A is a potent immunosuppressive agent which is widely used in the treatment of autoimmune diseases (26, 27). Both drugs, in theory, can be used in the treatment of PSS. This study showed that compared with the placebo response at the same baseline, the efficacy of belimumab and cyclosporine A administration were equivalent, which suggested that these two drugs had no significant therapeutic advantage in reducing the ESSDAI score. Thus, it is necessary to carefully determine the therapeutic value of these two drugs for PSS treatment.

However, this study had the following limitations: first, due to a high missing rate of race, height, and weight information of subjects in the literature, we failed to use them as covariates to investigate their effects on the placebo response; the time of PSS diagnosis as a covariate has some missing values. The results from the relevant literature (14) show that, in patients with symptoms for more than 5 years along with fibrosis of the exocrine glands, some symptoms may not be reversible, and these factors may lead to insufficient response to treatment. Second, the sample size of the placebo clinical trials for PSS is usually small (some may even include less than 10 subjects) and the lower sample size may contribute to large sampling errors and bias the results further. However, the results of the leave-one-out cross-validation (Jackknife) showed that small-sample trials had little effect on the model parameters (**Figure S4**), which indicated that the constructed placebo response model was robust. Third, the ESSDAI is currently used as an important outcome measure in clinical studies, however, there are some limitations to the ESSDAI. Using the ESSDAI, disease activity can be difficult to differentiate from damage. Since long-lasting features must not be scored, the ESSDAI declines with time even if the symptoms remain the same. And, some slight improvements in absolute values cannot be sufficient to cause changes in the ESSDAI domain category (39, 41). In addition, due to the paucity of the data, we failed to include the EULAR Sjogren’s Syndrome Patient Reported Index (ESSPRI), which aims to assess the subjective symptoms of patients. However, it has been pointed out (42) that the subjective (ESSPRI) and objective evaluation (ESSDAI) of PSS are not correlated. Finally, only English-language publications were included in this study, which may contribute to publication bias.

## Conclusion

In conclusion, using modeling, we quantitatively analyzed the time course and the factors influencing the placebo response (ESSDAI score) in pharmacotherapeutic clinical trials for PSS. It was found that the placebo response in primary Sjögren’s syndrome clinical trials showed a significant time course, which was significantly correlated with the ESSDAI score. This suggested that the impact of treatment duration and baseline heterogeneity between different studies need to be considered in future clinical trials for sample size estimation and cross-study comparisons. In addition, the placebo response established in this study provides a promising accurate external control for single-arm clinical trials and a valuable reference for drug development strategies and clinical practice.

## Data Availability Statement

The raw data supporting the conclusions of this article will be made available by the authors, without undue reservation.

## Author Contributions

L-JL and Q-SZ participated in conception and design of the work and revised the paper critically for important intellectual content. H-XL & Z-ZW selected studies and extracted the data, contributed to data reconciliation and cleaning. Z-ZW analyzed and interpreted the data, wrote the manuscript and revised the manuscript. All authors contributed to the article and approved the submitted version.

## Funding

This was not an industry supported study. This study was financially supported by the National Natural Science Funds (82174229). The authors declared no potential conflicts of interest with respect to the research, authorship, and/or publication of this article.

## Conflict of Interest

The authors declare that the research was conducted in the absence of any commercial or financial relationships that could be construed as a potential conflict of interest.

## Publisher’s Note

All claims expressed in this article are solely those of the authors and do not necessarily represent those of their affiliated organizations, or those of the publisher, the editors and the reviewers. Any product that may be evaluated in this article, or claim that may be made by its manufacturer, is not guaranteed or endorsed by the publisher.
