# Supplementary material for: Development and Application of the Placebo Response Model in Clinical Trials for Primary Sjögren’s Syndrome
Source: Front Immunol. 2021 Nov 16;12:783246. doi: 10.3389/fimmu.2021.783246 (PMC8635096; doi:10.3389/fimmu.2021.783246)
Supplement: Supplementary file 1 [file DataSheet_1.docx]

**Supplementary Material**

**Search strategy**

**Pubmed**

| No. | Query | Results | Date |
| --- | --- | --- | --- |
| #1 | “Placebos”[Majr] OR (Placebo[Title/Abstract]) OR (Sham Treatment[Title/Abstract]) | 228535 | 2021.5.28 |
| #2 | “Sjogren's syndrome”[Majr] OR (Sjogrens Syndrome[Title/Abstract])OR (Syndrome,Sjogren's[Title/Abstract])OR (Sjogren Syndrome[Title/Abstract])OR (Sicca Syndrome[Title/Abstract])OR (Syndrome, Sicca[Title/Abstract]) | 12550 | 2021.5.28 |
| #3 | #1 AND #2 | 131 | 2021.5.28 |
| #4 | #1 AND #2 Filters: Randomized Controlled Trial | 71 | 2021.5.28 |

**Embase**

| No. | Query | Results | Date |
| --- | --- | --- | --- |
| #1 | ‘Placebo’/exp OR Placebo | 481587 | 2021.5.28 |
| #2 | ‘Sjogren's syndrome’/exp OR ‘Sjogren's syndrome’ | 24968 | 2021.5.28 |
| #3 | ‘Sjogrens Syndrome’ OR(Sjogrens AND(‘Syndrome’/exp OR Syndrome)) | 553 | 2021.5.28 |
| #4 | ‘Syndrome, Sicca’ OR(Syndrome, AND Sicca) | 4113 | 2021.5.28 |
| #5 | #2 OR #3 OR #4 | 32409 | 2021.5.28 |
| #6 | #1 AND #5 | 603 | 2021.5.28 |
| #7 | #6 AND‘Randomized Controlled Trial’/de | 125 | 2021.5.28 |

**Cochrane Library**

| No. | Query | Results | Date |
| --- | --- | --- | --- |
| #1 | (“Sjogren's syndrome”)ti,ab,kw OR (“Sjogrens Syndrome”)ti,ab,kw OR (“Syndrome, Sjogren's”)ti,ab,kw OR (“Sjogren Syndrome”)ti,ab,kw OR (“Sicca Syndrome”)ti,ab,kw OR (“Syndrome, Sicca”)ti,ab,kw | 1282 | 2021.5.28 |
| #2 | (“Placebos”)ti,ab,kw OR (“Placebo”)ti,ab,kw OR (“Sham Treatment”)ti,ab,kw | 333434 | 2021.5.28 |
| #3 | #1 AND #2 Filters: Trials | 429 | 2021.5.28 |

**List of included studies**

1.Gottenberg J-E, Ravaud P, Puéchal X, et al. Effects of hydroxychloroquine on symptomatic improvement in primary Sjögren syndrome: the JOQUER randomized clinical trial. JAMA 2014;312:249–258.

2.Devauchelle-Pensec V, Mariette X, Jousse-Joulin S, et al. Treatment of primary Sjögren syndrome with rituximab: a randomized trial. Ann Intern Med 2014;160:233–242.

3.Bowman SJ, Everett CC, O'Dwyer JL, et al. Randomized controlled trial of rituximab and cost-effectiveness analysis in treating fatigue and oral dryness in primary Sjögren's syndrome. Arthritis Rheumatol 2017;69:1440–1450.

4.St Clair EW, Baer AN, Wei C, et al. Clinical efficacy and safety of Baminercept, a lymphotoxin β receptor fusion protein, in primary Sjögren's syndrome: results from a phase II randomized, doubleblind, placebo-controlled trial. Arthritis Rheumatol 2018;70:1470–1480.

5.Thomas Dörner,Maximilian Georg Posch,Yue Li, et al. Treatment of primary Sjögren’s syndrome with ianalumab (VAY736) targeting B cells by BAFF receptor blockade coupled with enhanced, antibody-dependent cellular cytotoxicity. Annals of the Rheumatic Diseases 2019;78: 641–647.

6.Juarez M,Diaz Nieves,Johnston G,et al. A phase 2 randomized, double-blind, placebo-controlled, proof-of-concept study of oral seletalisib in primary Sjögren's syndrome. Rheumatology (Oxford, England) 2020.

7.Posada J,Valadkhan S,Burge D,et al. Improvement of Severe Fatigue Following Nuclease Therapy in Patients With Primary Sjögren’s Syndrome: A Randomized Clinical Trial. Arthritis & Rheumatology 2020;73(1).

8.Van Nimwegen J.F,Mossel, E,van Zuiden G.S,Wijnsma,et al. Abatacept treatment for patients with early active primary Sjögren’ssyndrome: A single-centre, randomised, double-blind, placebo-controlled, phase 3 trial (ASAP-III study).Lancet Rheumatol 2020;2:e153–e163.

9.van der Heijden EHM,Sofie Liny Marie Blokland,Maarten Reinier Hillen,et al. Leflunomide–hydroxychloroquine combination therapy in patients with primary Sjögren's syndrome (RepurpSS-I): a placebo-controlled, double-blinded, randomised clinical trial The Lancet Rheumatology 2020;2(5).

10.Benjamin A Fisher,Antonia Szanto,Wan-Fai Ng,et al. Assessment of the anti-CD40 antibody iscalimab in patients with primary Sjögren's syndrome: a multicentre, randomised, double-blind, placebo-controlled, proof-of-concept study. The Lancet Rheumatology 2020;2(3).

11.Baer Alan N,Gottenberg JE,St Clair E William,et al. Efficacy and safety of abatacept in active primary Sjögren's syndrome: results of a phase III, randomised, placebo-controlled trial. Annals of the rheumatic diseases 2020.

12.Felten Renaud,Devauchelle-Pensec V,Seror Raphaèle,et al. Interleukin 6 receptor inhibition in primary Sjögren syndrome: a multicentre double-blind randomised placebo-controlled trial. Annals of the Rheumatic Diseases 2020.

**Covariate screening process**

$P_{i}=P_{Typical}+(COV-COV_{median})\times\theta_{\mathrm{cov}}+\eta_{i}$ (4)

$P_{i}=P_{Typical}\times(COV/COV_{median})^{\theta_{\mathrm{cov}}}+\eta_{i}$ (5)

$P_{i}=P_{Typical}+COV\times\theta_{\mathrm{cov}}+\eta_{i}$ (6)

Continuous covariates on the parameters were modeled according to Eqs. 4 and 5. Equation 6 tested the influence of categorical covariates on the parameters. As seen in Eqs. 4 to 6, P_i_ is a pharmacodynamic parameter value of the i^th^ placebo group, P_Typical_ is the typical value of a pharmacodynamic parameter of the overall placebo group, COV is the covariate value of the i^th^ placebo group, COV_median_ is the median value of the covariate, and θ_cov_ is the correction coefficient of the covariates on the pharmacodynamic parameter. η_i_ is an inter-group variability of a pharmacodynamic parameter. During the covariate model building process, a reduction in objective function values (OFV) of 3.84 and 6.63 were considered significant at P = 0.05 and P = 0.01, respectively, for a nested model with one degree of freedom difference. All covariates were analyzed in a step-wise manner with a forward selection step (P = 0.05) and a stricter backward elimination step (P = 0.01).

**Table S1.** Summary of the included studies(only for placebo group)

| Author，year | Type | Sample size | Treatment | Duration  (week) | Trial location | Age  (year) | ESSDAI Baseline  (score) | Male  (%) | Time to diagnosis  (year） |
| --- | --- | --- | --- | --- | --- | --- | --- | --- | --- |
| Gottenberg^28^ 2014 | RCT | 64 | Hydroxychloroquine | 24 | France | 55.6 | 2.5 | 6.2 | 1 |
| Devauchelle-Pensec^29^  2014 | RCT | 57 | Rituximab | 24 | France | 55.6 | 10.2 | 3.5 | 5.5 |
| Bowman^30^ 2017 | RCT | 66 | Rituximab | 48 | U.K. | 54.4 | 6 | 7.6 | 6.2 |
| St Clair^31^ 2018 | RCT | 19 | Baminercept | 24 | America | 55 | 3.8 | 5 | NA |
| Dorner^32^  2019 | RCT | 9 | ianalumab | 24 | Switzerland | 50 | 10 | 22.2 | NA |
| Juarez^33^  2020 | RCT | 14 | seletalisib | 12 | International | 60.2 | 13.1 | 7.1 | 7.6 |
| Posada^34^ 2020 | RCT | 8 | RSLV-132 | 14 | U.K. | 59.6 | 5.4 | 0 | NA |
| Van Nimwegen^35^ 2020 | RCT | 40 | Abatacept | 24 | Netherlands | 49 | 13 | 7 | 2 |
| Vander Heijden^36^ 2020 | RCT | 8 | Leflunomide  –hydroxychloroquine | 24 | Netherlands | 53.5 | 9.1 | 0 | 8.9 |
| Fisher^37^ 2020^1^ | RCT | 11 | iscalimab | 12 | International | 50.6 | 11 | 0 | NA |
| Fisher^37^ 2020^2^ | RCT | 4 | iscalimab | 12 | International | 48.8 | 11.8 | 0 | NA |
| Alan N Baer^38^  2020 | RCT | 95 | Abatacept | 24 | International | 52.9 | 10.1 | 3.2 | 5.1 |
| Renaud Felten^39^ 2020 | RCT | 55 | tocilizumab | 44 | France | 54.8 | 12.2 | 9.1 | 4.9 |

NA: not reported.

**（A）**


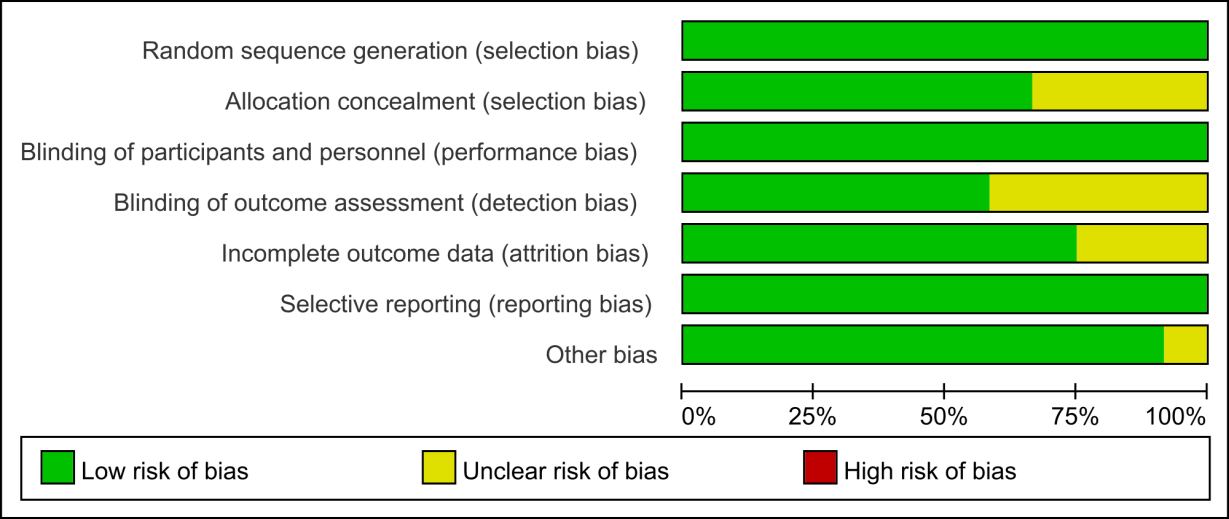
**
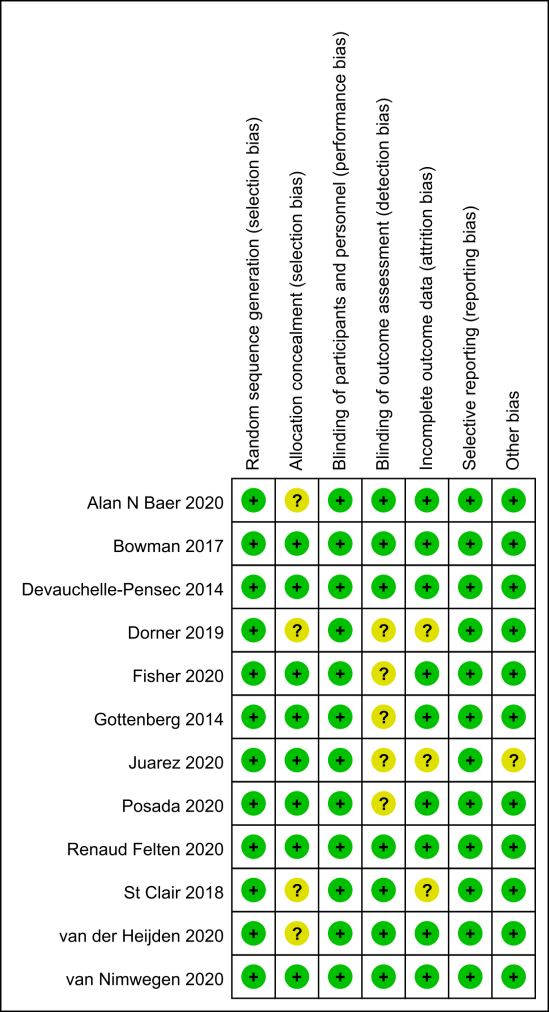
**

**(B)**

**Figure S1.** Risk of bias assessment chart.Using Cochrane’s risk of bias assessment tool to assess the Study-level (A) and Overall (B) risk of bias. In this tool, studies were deemed to be at high, low or unclear risk of bias based on adequacy of sequence generation, allocation concealment, blinding, processing of incomplete data, selective reporting, and other biases.

**(A)**


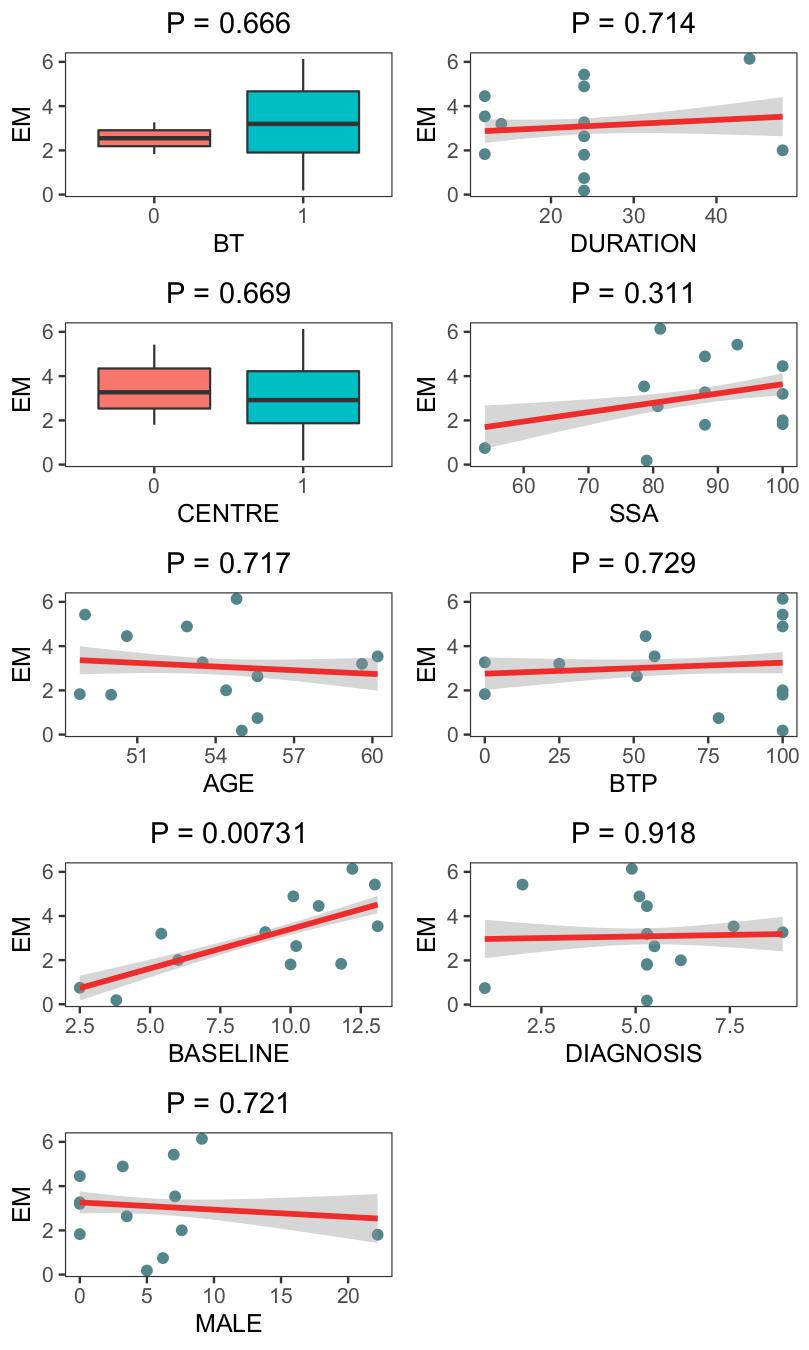


**（B）**


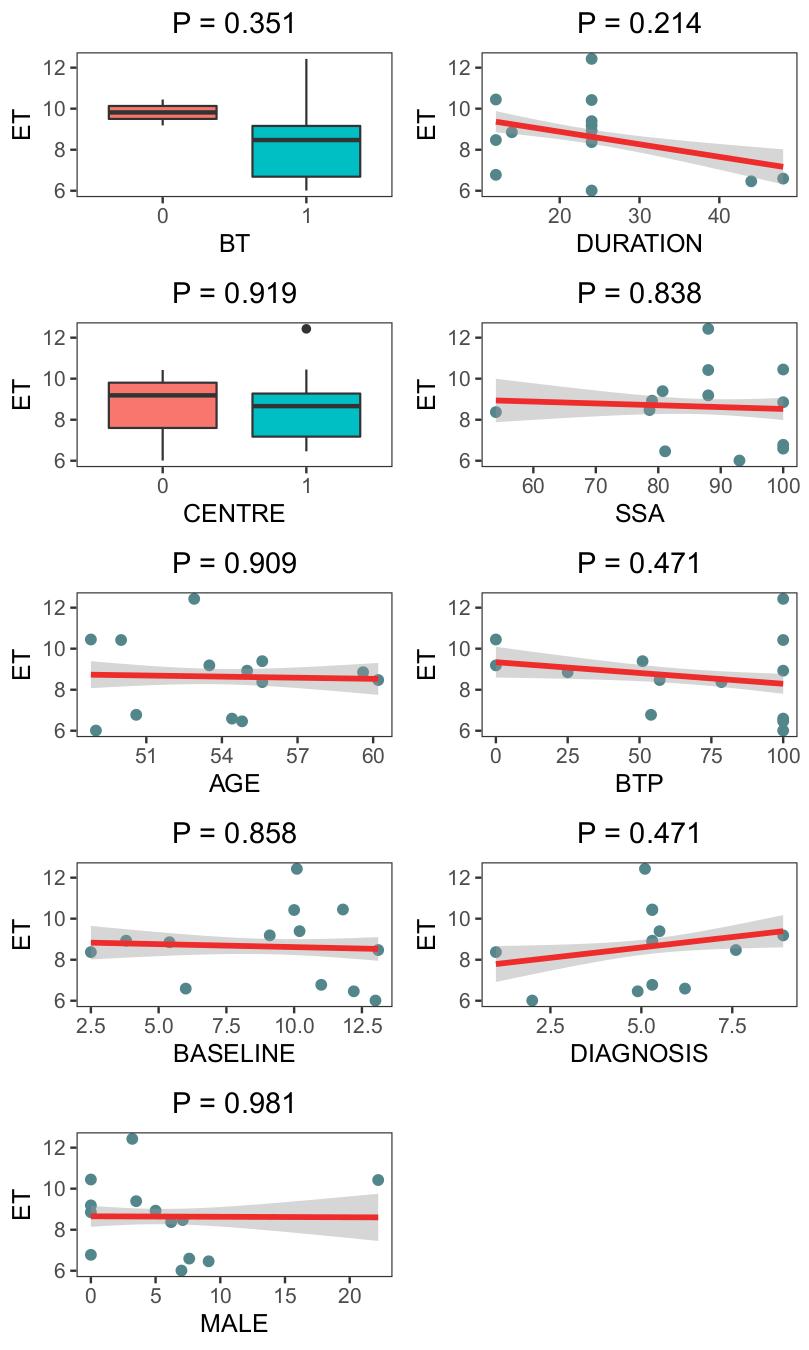


**Figure S2.** The relationship between the covariates and the pharmacodynamic parameters E_max_ (A) and ET_50_ (B). In the sub-graph of BT, 1 represents subjects with basic therapy, and 0 represents subjects with no basic therapy;In the sub-graph of Centre, 1 represents multicenter, and 0 represents singlecenter;In the sub-graph of SSA, the value of the abscissa represents the proportion of anti-SSA positive;In the sub-graph of BTP, the value of the abscissa represents the proportion of subjects with basic therapy;In the sub-graph of diagnosis, the value of the abscissa represents the disease diagnosis time;In scatter plots, the solid line represents the trend line and the shaded area represents the 95% CI of the trend line.


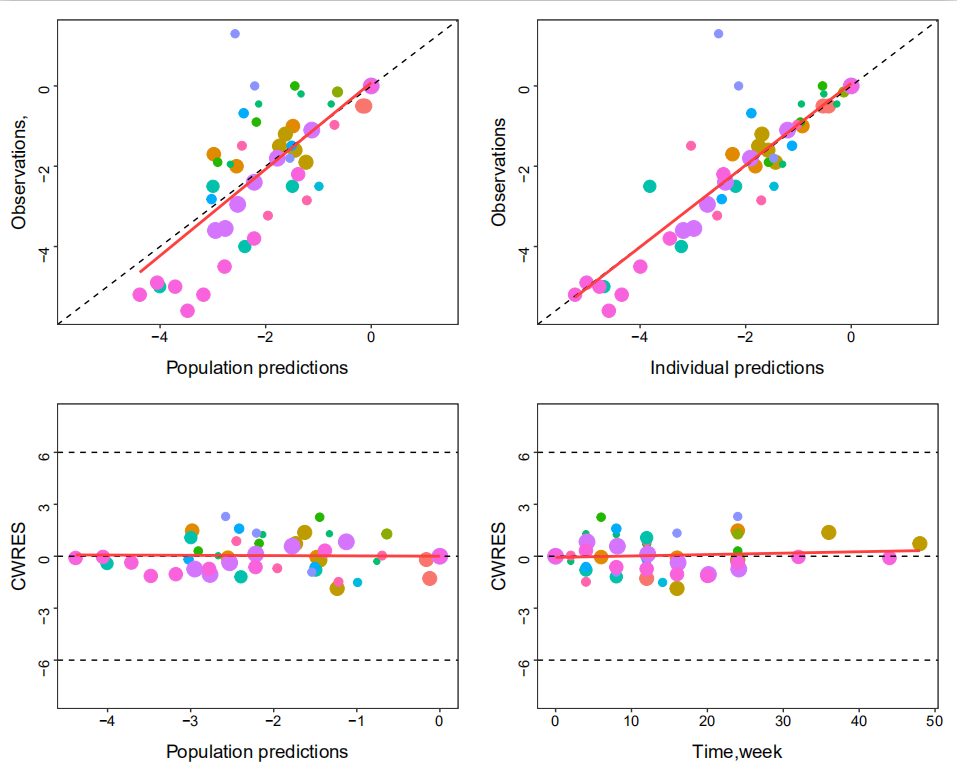


**Figure S3.** Goodness-of-fit plots for the final model. The upper left, Population predictions versus observations.The upper right,Individual predictions versus observations.The lower left, Conditional weighted residuals(CWRES) versus population predictions.The lower right,Conditional weighted residuals(CWRES) versus time. Point size is related to sample size, and the solid red line represents the fitting line. The dashed black lines in the upper left and right panels are diagonal, and the dashed black lines in the lower left and right panels are the 0 line and the ± 6 reference line, respectively.


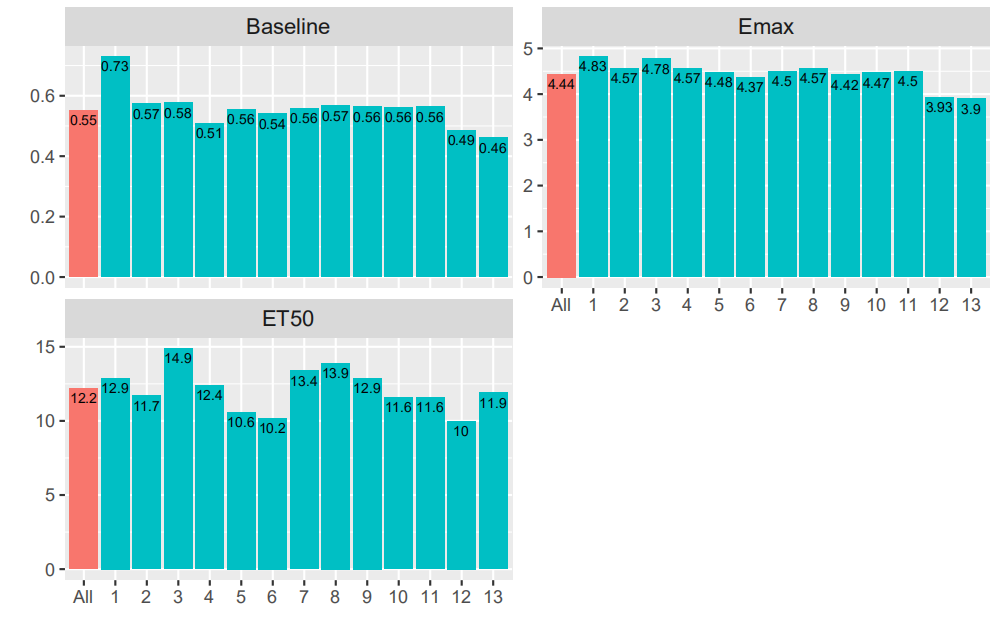


**Figure S4.** Charts of the typical parameter values obtained from the ﬁnal model in a leave-one-out cross validation (Jackknife).The abscissa represents the study number dropped from the full data and the ordinate, the typical value of parameters.The left red bar represents parameters obtained from full data.
